# A U-Net Improved Version for Crop and Weed Segmentation from Aerial Images

**DOI:** 10.3390/s26102997

**Published:** 2026-05-09

**Authors:** Alexandru Bunica-Mihai, Dan Popescu, Loretta Ichim

**Affiliations:** Faculty of Automatic Control and Computers, National University of Science and Technology POLITEHNICA Bucharest, 060042 Bucharest, Romania; alexandru.bunica@stud.acs.upb.ro (A.B.-M.); loretta.ichim@upb.ro (L.I.)

**Keywords:** artificial neural networks, ensemble of neural networks, image segmentation, crops, weed identification

## Abstract

The optimization of herbicide application is one of the most important topics in Precision Agriculture, driven by both economic efficiency and ecological sustainability. Excessive herbicide use can lead to soil degradation, water contamination, and negative impacts on biodiversity, while also contributing to human health risks and climate-related concerns. Developing accurate, automated approaches for distinguishing crops from weeds is therefore essential to support sustainable agricultural practices. In this paper, a novel architecture for crops and weed segmentation in tobacco plantations is proposed: a U-Net variant which incorporates several specific design elements, including deep supervision, a Vegetation Global Context block, and a dual-headed output that separately predicts vegetation and crop masks. Weed regions are derived as the difference between vegetation and crop predictions, allowing the model to enforce logical consistency directly within a single framework, in contrast to other two-step approaches. The proposed architecture was evaluated using multiple modern encoder backbones (ConvNextV2, FastViT, RepViT, MambaVision). Experimental results demonstrate that this architecture not only improves segmentation accuracy compared to prior approaches, with best scores of 94.24% Dice for crop segmentation and 93.72% for weeds, but also significantly reduces inference time by avoiding multi-stage pipelines, making it well-suited for real-time deployment.

## 1. Introduction

In the domains of Smart and Precision Agriculture, the detection and segmentation of weeds represent an important step in the adaptive application of pesticides, one of the most important tasks in an increasingly more pressing need for environmentally responsible, resource-efficient, and economically sustainable agricultural practices. With the steady growth of the global population and the parallel intensification of agricultural practices, the indiscriminate use of herbicides has led to severe environmental, economic, and health-related concerns. Reports indicate a projected increase in global food demand by an estimated 50–60% between 2010 and 2050 to meet the needs of a larger and more affluent population, placing additional pressure on agricultural systems to produce more with limited resources while avoiding further ecosystem degradation [1]. Both rising global population and increasing per capita income significantly drive the demand for food—especially for calories and for higher-value food products such as meat and dairy, which require more crop calories as feed. Under a central scenario with a 39% population increase by 2050, total available food calories are projected to grow by about 44%, and crop calories by about 47% relative to 2011. Future increases in food demand will continue to exert pressure on cropland and agricultural systems. How this demand is met—as a function of productivity growth, dietary change, and land use adjustment—affects food prices, resource use, and the size of the global agricultural footprint by mid-century [2]. While the agricultural land area has also consistently increased through the years, and agricultural productivity growth has reduced the use of natural and environmental resources, the total factor productivity growth has slowed down between 2011 and 2020, and this stagnation may affect food prices, the expansion of agriculture into more natural lands, and global food security [3]. The effects of climate change on agricultural activity and yields may further affect the capacity of food systems to meet rising demand, as increasing temperatures, altered precipitation patterns, and more frequent extreme weather events are projected to reduce average yields for major staple crops in many regions and place additional stress on production systems. Under severe climate scenarios and without effective adaptation, scientists indicate simulated losses in crop yields such as wheat, rice, and maize range from approximately 7% to 23%, highlighting the sensitivity of global agriculture to climatic shifts and water stress [4]. Widespread and repeated herbicide applications have been linked to contamination of soil and aquatic systems via runoff and leaching, reductions in plant, microbial, and animal biodiversity, and the proliferation of herbicide-resistant weed populations that complicate management and ecosystem function. These impacts include adverse effects on non-target organisms across trophic levels, alterations in community structure, and diminished habitat quality in agroecosystems [5]. Precision Agriculture addresses these challenges by tailoring field treatments to local conditions, reducing chemical usage while maintaining or improving crop yields.

Within this framework, accurate weed detection and segmentation enable site-specific weed management strategies, such as variable-rate spraying [6,7], robotic herbicide application [8] and robotic mechanical removal [9,10]. These approaches rely heavily on computer vision and machine learning techniques capable of distinguishing crops from weeds under highly variable field conditions, including changes in illumination, soil background, crop growth stages, and weed morphology. Deep learning approaches, such as convolutional neural networks, vision Transformers, and hybrids, have revolutionized agricultural image analysis, making it possible to identify and map weeds in real time with high spatial accuracy. By learning rich visual features directly from field images, these networks overcome the limitations of manually designed descriptors and remain robust to environmental variability. Fully convolutional and attention-based architectures provide detailed, pixel-level segmentation, enabling precise weed localization and supporting downstream actions such as variable-rate spraying or robotic removal. These advances position deep learning as a key technology for efficient and scalable site-specific weed management [11].

Nevertheless, several challenges remain. The high intra-class variability of weeds, their visual similarity to crops at early growth stages, and the scarcity of well-annotated datasets limit the robustness and generalizability of existing models. Furthermore, deployment constraints such as computational efficiency, energy consumption, and real-time inference requirements must be considered for practical field applications [12]. Addressing these issues is crucial for the development of reliable, scalable, and environmentally responsible weed control systems, reinforcing the central role of weed detection and segmentation in the broader context of sustainable and intelligent agricultural practices.

The ultimate purpose of models capable of accurately distinguishing between crops and weeds, with precise localization and surface estimation, may be the integration of such architectures into broader, scalable monitoring platforms, where they will no longer be isolated tools, but components within a larger intelligent pipeline. Recent advancements in areas like IoT, Big data, and Cloud computing have spurred an all-new interest in integrated, data-driven agricultural ecosystems, where real-time insights can be continuously collected, processed, and acted upon [13,14]. For example, the authors in [15] present a complex software solution that aims to support decision-making through real-time and predictive analytics by using a layered architecture, which goes from the IoT device layer, which collects data from multiple sensor sources, such as soil and weather sensors, or UAV-mounted optical sensors, to a cloud computing layer, for advanced analytics and storage. The platform includes an application marketplace, which supports the creation of an extensible, modular plugin system that allows users to create and add tools for specific tasks. CloudCropFuture [16] is another recently proposed scalable platform for greenhouse monitoring, which can integrate different models for different tasks, such as disease detection, maturity assessment, and crop quality evaluation. Each of the subsystems for these tasks uses data that is collected through an acquisition and monitoring system, which includes high-precision sensors for environmental data and imaging. The sensor data is then used by the edge “Environmental Monitoring Host”, which communicates with the cloud-based server. Data-driven commands may be given by a human operator through a mobile app or web interface, or be automatically decided by the Host, which actuates different execution nodes, such as irrigators or ventilation systems. Another interesting direction is the creation of Digital Twin models, which may simulate agricultural activities to predict various outcomes, such as yields or diseases. In [17], an edge-cloud architecture is employed for creating a virtual model that simulates the scene of a tomato farm. An edge layer collects data from multiple sensors, such as cameras, and preprocesses them, while the cloud layer stores this information and runs object detection models and data transmission services. The application layer builds a virtual 3D farm model and allows the user to visualize and interact with the data.

It becomes evident that the importance of sensors in such platforms is critical. A wide variety of sensors may be employed, ranging from environmental sensors that monitor soil moisture, temperature, humidity, and light intensity, to imaging sensors such as RGB, multispectral, hyperspectral, or thermal infrared cameras, which provide detailed visual information about crop conditions [18]. Beyond direct visual observation, such imaging modalities also enable the extraction of biochemical and physiological indicators that are highly relevant for crop–weed discrimination. Vegetation indices, such as the Normalized Difference Vegetation Index (NDVI), exploit differences in spectral reflectance to capture variations in chlorophyll content and plant vigor. These differences can be leveraged to distinguish crops from weeds, as they often exhibit distinct growth patterns, stress responses, and biochemical signatures [19].

Tobacco (*Nicotiana tabacum* L.) is the principal species cultivated for commercial tobacco production worldwide, valued for its cured leaf used in smoking, chewing, and nicotine extraction. Though tropical in origin, cultivated tobacco is grown across a wide range of climates and requires a frost-free period following transplanting to reach maturity in the field, with seedlings typically raised in beds and later transplanted at defined spacings into prepared soil [20]. The crops’ establishment as transplants and relatively wide row spacings expose considerable bare soil early in the season, which can facilitate weed emergence that competes with young plants for light, water, and nutrients, making precise and timely weed management a key agronomic practice in tobacco systems.

Weed infestations in tobacco fields are diverse and highly variable, consisting of both annual and perennial species with growth patterns that often overlap with the crop during early phenological stages, leading to significant crop losses [21]. Many common tobacco weeds exhibit morphological similarities to young tobacco plants, complicating visual discrimination and increasing the risk of misapplication during control operations. Additionally, tobacco is particularly sensitive to herbicide injury, which limits the range and dosage of chemical treatments that can be safely applied [22]. These factors make conventional blanket spraying inefficient and potentially damaging, highlighting the need for accurate weed detection and segmentation methods that enable selective interventions tailored to the specific weed pressure within tobacco fields.

In this context, the following paper presents a hierarchical semantic segmentation solution that accurately distinguishes crops from weeds in tobacco fields, leveraging multi-scale contextual information and spatially aware decoding to capture fine-grained structures while maintaining efficiency suitable for real-world field applications.

Numerous works on weed detection and segmentation expand on methods based on either encoder–decoder architectures or forms of Pyramid Pooling. Building on these foundations, recent research increasingly focuses on integrating multi-scale attention mechanisms and lightweight backbones to balance precision with real-time performance in the field. Some approaches fuse spectral or depth cues with RGB imagery to mitigate occlusion and illumination variability, while others explore transformer-based designs that capture broader spatial context without sacrificing fine-grained boundary detail. Together, these directions reflect a shift toward models that can generalize across diverse crop types, growth stages, and environmental conditions while remaining efficient enough for deployment on edge devices in agricultural settings.

To enable deployment on edge devices and UAVs, significant effort has been directed toward reducing the computational burden of semantic segmentation models. Zuo and Li [23] proposed an improved U-Net by replacing the standard encoder with MobileNetV3 and integrating a Pyramid Pooling Module (PPM), achieving high accuracy with reduced parameters in weed segmentation in corn fields. Similarly, the authors in [24] introduced CWRepViT-Net, which utilizes RepViT blocks in the encoder and a modified U-Net decoder, leveraging transfer learning to balance speed and precision. Habib et al. [25] proposed DWUNet, which employs blocks centered around depth-wise separable convolutions to minimize inference time (8 ms per image).

Beyond CNNs, lightweight Transformers have also gained traction. Castellano et al. [26] adapted Lawin, a Transformer-based encoder–decoder semantic segmentation architecture, for weed mapping by adding NIR and RE bands capability through the addition of a second encoder and multiple feature fusion blocks. In [27], the authors propose an architecture with an encoder akin to Segformer’s Mix Transformer [28], with overlapped patch merging, efficient self-attention, and Mix-FFN. The decoder employs a two-branch approach: after the multi-scale encoder features are passed through a Spatial MLP block, the Dynamic Context Aggregation and Local Detail Enhancement branches model the global contextual information and the local details, respectively. The features from the two parallel branches are then fused and processed through multiple Spatial MLP blocks.

To address the limitations of CNNs in capturing global context and Transformers in capturing local details, recent works have increasingly adopted hybrid architectures. Jiang et al. proposed SWFormer [29], a scale-wise hybrid network that integrates Convolutional Modulation with Transformer blocks to capture multi-granularity information. Sun et al. [30] developed a dual-branch architecture combining a CNN branch for local boundary enhancement and a Transformer branch for global modeling. Similarly, Madeshwar et al. [31] and Mei et al. [32] integrated attention mechanisms (AMFF—Adaptive Multispectral Feature Fusion—and AFMA—Across Feature Mapping Attention—, respectively) into U-Net-like structures to improve the segmentation of small, irregular weed targets. While these architectures improve accuracy, they process the entire image at high resolution or high complexity, which can be computationally wasteful, especially for images with large, simple background areas.

Recognizing that single-stage models often struggle with fine details, hierarchical approaches have been proposed to refine predictions. Awedat [33] introduced a dual-stage framework that first detects weeds using YOLOv8 (Object Detection) and then refines the bounding box regions using U-Net (Segmentation). This “detect-then-segment” approach restricts expensive segmentation to relevant areas but relies on the assumption that weeds fit neatly into bounding boxes, which is often not the case for amorphous weed patches.

Other hierarchical methods focus on resolution rather than selection. Zhao et al. [34] combined Super-Resolution Reconstruction (SRR) with semantic segmentation to enhance low-resolution drone imagery before processing. A similar work was presented in [35]. Cheong et al. [36] addressed field-of-view limitations by using an “outpainting” teacher network to guide a student network. These methods generally apply refinement globally or based on spatial constraints, rather than model confidence. Uncertainty estimation has also been explored to increase the reliability of agricultural robotic systems. In [37], Celikkan et al. proposed a Bayesian DeepLabv3+ that outputs pixel-wise uncertainty estimates using Monte Carlo Dropout. Their work demonstrates that uncertainty maps can effectively highlight areas where the model is prone to error (e.g., boundaries and occlusions).

The authors in [38] propose a two-step approach that most closely resembles our method. Their work demonstrates that weed segmentation can be simplified by first addressing the easier task of segmenting overall vegetation. In the first stage, the model performs binary segmentation, classifying pixels as either vegetation or background, with background pixels set to zero. The resulting output is then used as input for a three-class segmentation task, distinguishing background, crop, and weed, with better scores than a traditional, one-stage U-Net. Our own past work [39] refined this concept using an EfficientNet-backed two-stage U-Net, in which both stages performed binary segmentation, achieving improved overall results, both in terms of accuracy and speed. While effective, the sequential nature of these approaches increases computational cost and latency.

Despite these advancements, a critical disconnect remains between architectural efficiency and hierarchical feature discrimination. Existing lightweight solutions (e.g., MobileNet-based U-Nets) achieve inference speed by significantly reducing network depth, often sacrificing the high-level semantic capacity required to distinguish morphologically similar crops and weeds. Conversely, heavier architectures or Transformer-based models offer superior segmentation fidelity but are computationally prohibitive for real-time edge deployment. Furthermore, current methodologies still struggle to handle the geometric irregularity of agricultural targets. Multi-stage approaches, often times slower, also tend to rely on object detection priors (bounding boxes), which are ill-suited for amorphous, sprawling weed patches. While attention mechanisms have been proposed to address this, they are rarely integrated effectively with modern hierarchical backbones in a way that enforces logical consistency across scales and are most often computationally expensive.

To address these limitations, we propose a single-stage segmentation network that integrates multi-scale contextual reasoning, simple spatial attention, and a Vegetation Global Context module. By producing separate vegetation and crop predictions in one forward pass, the network derives the weed probability as the residual vegetation not explained by crops, enforcing logical consistency between classes. Multi-scale deep supervision guides intermediate features, enabling precise delineation of fine-grained structures while maintaining computational efficiency suitable for real-time deployment. We experiment with several modern backbones to illustrate how different architectures balance segmentation performance and inference speed.

## 2. Materials and Methods

### 2.1. Dataset

**Figure 1:**
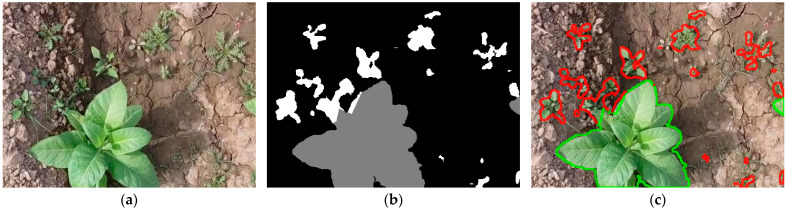
Example of a patch-mask pair in the dataset. (**a**) a 480 × 352 patch; (**b**) ground-truth segmentation mask for the patch. The gray areas represent tobacco plants, and the white areas represent weeds; (**c**) patch with the ground-truth mask’s edges overlaid (green for tobacco, red for weeds).

**Figure 2:**
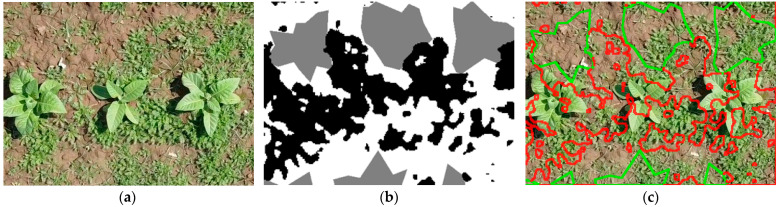
Example of a mismatched (**a**) patch image and (**b**) mask pair in the dataset (101.png from Campaign no. 1), with (**c**) showing the patch with the overlaid mask edges to highlight the misalignment (green for tobacco, red for weeds).

The dataset used for the experiments in this paper was introduced in [38], and is publicly available at [40]. It comprises 210 images, taken across eight campaigns in Mardan, Khyber, Pakhtunkwa, Pakistan, using a Mavic Mini drone, at an average altitude of 4 m and a resolution of 1920 × 1080. This drone employs a 1/2.3″ CMOS sensor camera, which can shoot up to 2.7 K video and 12 MP photos, with an aperture of f/2.8. It also includes a 3-axis gimbal for stabilization [41]. The authors also provide 480 × 352 resolution patches extracted from the sensor data. The segmentation masks were drawn manually and included three classes: background (pixel value of 0), tobacco plant (value of 1) and weed (value of 2). An example of such a patch is presented in Figure 1. Unfortunately, the dataset presents two major issues. First, there are inconsistencies between the masks and the patches: files sharing the same name do not correspond to the same region of the original image. We have found that this is only a misnaming problem. A case like this is illustrated in Figure 2.

**Figure 3:**
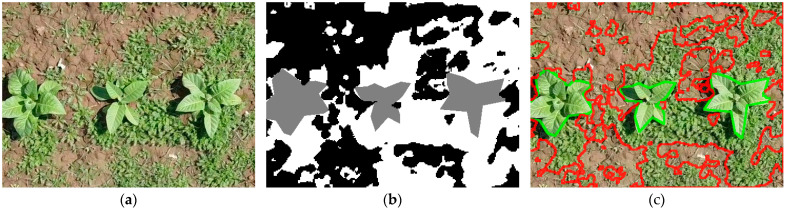
(**a**) The same original image as in Figure 2; (**b**) its correct ground-truth mask, found using the k-nearest neighbors HSV filter approach in the same dataset and (**c**) the patch with the ground-truth mask’s edges overlaid (green for tobacco, red for weeds).

To address this issue, we extracted binary masks using an HSV-based green filter. For each image patch, the extracted mask was compared against the set of ground truth masks in the dataset using the k-nearest neighbors (k-NN) algorithm. The ground truth mask with the smallest distance to the extracted mask was identified as the corresponding match, and the masks were renamed to ensure correct correspondence. Figure 3 shows the correct pair found in the same database for the mismatched image above. Note that this process did not in any way modify the ground-truth masks themselves.

The second issue concerns the noise present in the masks from the second data acquisition campaign. Our preliminary experiments showed that these noisy samples negatively impact both the segmentation models and the evaluation process. Consequently, we decided to exclude this portion of the dataset from our experiments.

### 2.2. Neural Networks Used

#### 2.2.1. U-Net Architecture

To effectively capture both semantic context and local textural details, we employ a U-Net architecture. The network follows a symmetric encoder–decoder design. The encoder (contracting path) progressively reduces the spatial resolution of the input while increasing feature dimensionality, enabling the extraction of abstract semantic representations. The decoder (expanding path) restores spatial resolution through successive upsampling stages, combining high-level features with corresponding encoder features via skip connections to preserve localization accuracy.

Building upon the standard U-Net formulation, we introduce multiple specialized modifications. These include a Vegetation Global Context block, designed to integrate global contextual cues relevant to vegetation structure, and a dual-headed output design that predicts vegetation and crop masks separately. This formulation allows weed regions to be computed hierarchically as the difference between vegetation and crop predictions, enforcing logical consistency directly within the network.

We also experimented with multiple modern encoder backbones within the same U-Net framework: ConvNeXt V2, FastViT, RepViT and MambaVision. These include convolutional, hybrid convolution–transformer, and state-space–inspired models.

#### 2.2.2. ConvNeXt V2

Introduced in [42] as an attempt to apply Vision Transformer concepts in building a ConvNet architecture, ConvNeXt modernized standard ResNets, enabling them to compete with the emerging state-of-the-art transformer-based models. By adopting design principles from transformers—such as larger kernel sizes, inverted bottlenecks, and simplified normalization—ConvNeXt retained the efficiency and inductive biases of convolutions while achieving improved accuracy.

The architecture employs a minimalistic design: it replaces the original ResNet stem and bottleneck blocks with streamlined convolutional blocks, uses LayerNorm instead of BatchNorm, and incorporates depthwise convolutions to capture long-range dependencies more effectively. ConvNeXt has demonstrated strong performance on image classification benchmarks, rivaling that of Vision Transformers while maintaining lower computational complexity and better scalability for downstream tasks such as object detection and segmentation. The paper presents multiple variants—Tiny, Small, Base, and Large—which differ in the number of channels and layers per stage, scaling model capacity and computational cost.

An updated version of the architecture, ConvNeXt V2, was proposed in [43]. Building on the strengths of its predecessor, ConvNeXt V2 adopts an adapted version of the masked autoencoder self-supervised training strategy, in which random portions of the input image are masked, and the model is trained to reconstruct the missing content from the visible context using an encoder–decoder framework.

This pretraining approach encourages the network to learn richer and more generalizable visual representations, improving data efficiency and robustness. In addition, ConvNeXt V2 introduces architectural refinements such as Global Response Normalization (GRN), which enhances feature competition and stabilizes training at scale. Together, these changes allow ConvNeXt V2 to achieve state-of-the-art performance across both supervised and self-supervised settings, while preserving the simplicity and efficiency characteristic of convolutional networks.

**Figure 4:**
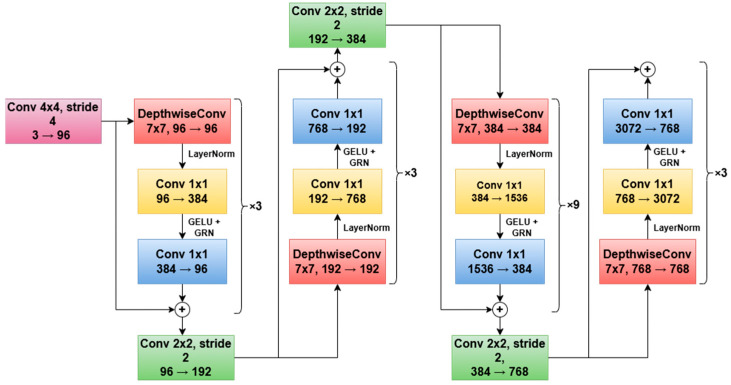
The four stages of the ConvNeXt V2 Tiny architecture, including the initial stem and intermediary downsampling steps.

Figure 4 illustrates the four stages of the ConvNeXt V2 Tiny architecture, including the first stem convolution and the downsampling steps, which mark the transitions between them. Each stage corresponds to a repetition of a block, which consists of a depthwise convolution and two pointwise convolutions (the red, yellow, and blue squares). The accolades suggest that each block is repeated an ×k number of times before the downsampling. The values below each convolution show the evolution of the number of channels.

#### 2.2.3. FastViT

Designed to achieve very low latency while retaining strong representational capacity, FastViT [44] is a hybrid vision transformer model that combines re-parameterized convolutional blocks with lightweight token-mixing mechanisms. The architecture is based on the principle of structural re-parameterization, in which multi-branch training-time structures are merged into single convolutional layers at inference, eliminating the need for explicit skip connections. It comprises four hierarchical stages with progressively decreasing spatial resolution and increasing feature dimensionality. Within each stage, feature mixing is achieved through depthwise convolutions that enable efficient re-parameterization of residual pathways. In the final stage, conventional self-attention is replaced by large-kernel convolutions, reducing computational complexity while retaining a wider, non-local receptive field.

The model also replaces all dense k × k convolutions with their factorized versions, akin to depthwise separable convolutions. While this leads to better efficiency, it also lowers the parameter count, which may reduce performance. To compensate, the architecture overparametrizes those replaced layers, found in the convolutional stem, patch embedding, and projection layers, at train-time.

#### 2.2.4. RepViT

Proposed in [45] as a modernization of MobileNets by incorporating aspects of the Vision Transformer’s architectural design, RepViT also focuses on convolutional mixing and structural reparametrization. By moving the depthwise convolution up, in a separate branch to the 1×1 expansion convolution. This effectively separates, as in the case of ViT, token (spatial) and channel mixing. The formed skip connection is omitted at inference through reparametrization. The expansion ratio is fixed to 2 throughout the whole network, rather than the variable 2, 3, and 6 of MobileNetV3, and further optimizations are made for mobile devices, such as simplifying the early stem convolutions, deepening the downsampling block, simplifying the final classifier, redistributing the stage ratio for deeper late stages, and removing the 5 × 5 convolutions and repositioning squeeze-and-excitation modules to appear only once every two blocks. These modifications collectively reduce parameter count and computational cost while maintaining expressive power and a large effective receptive field.

#### 2.2.5. MambaVision

Combining Conv-Transformer hybrids with Mamba state space models, the MambaVision architecture achieved SOTA performance on the ImageNet-1K dataset by introducing the MambaMixer block in another four-stage network, which leverages structured state-space layers to model long-range spatial dependencies efficiently, integrates local convolutional processing for fine-grained feature extraction, and incorporates self-attention in deeper stages to capture global context. The original Mamba mixer specializes in vision tasks, replacing causal convolution with regular convolution and adding a parallel branch with no state-space modeling to capture spatial information.

All encoder backbones were initialized with ImageNet-pretrained weights and integrated without architectural modifications to the proposed decoder, ensuring that observed differences reflect encoder characteristics rather than changes in the segmentation architecture.

### 2.3. Proposed Architecture

**Figure 5:**
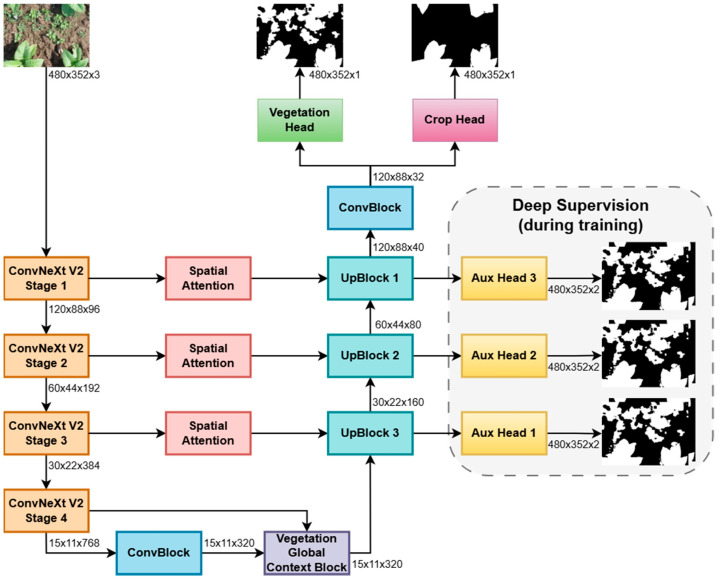
The proposed U-Net segmentation architecture with a ConvNeXt V2 backbone.

The proposed architecture, with a ConvNeXt V2 encoder, is presented in Figure 5. The ConvBlocks consist of two consecutive 3 × 3 convolutional layers, each followed by a ReLU activation. The decoder comprises three upsampling blocks (UpBlock). Each block performs bilinear upsampling, applies Spatial Attention to the corresponding skip connection, concatenates the features, and refines them with a convolutional block. For supervision, both the auxiliary heads and the final heads produce logits at the spatial resolution of their respective decoder outputs. These logits are then bilinearly interpolated to match the input image dimensions before loss computation. This ensures that all predicted masks—whether intermediate or final—are directly comparable to the ground-truth annotations at full resolution, enabling coherent multi-scale learning.

The model employs two separate output heads, one for vegetation and one for crops, each implemented as a simple 1 × 1 convolution, allowing the network to learn specialized features for each class. This separation facilitates hierarchical reasoning, as weeds can be derived from the difference between the vegetation and crop masks, and helps enforce logical consistency in the segmentation while improving overall accuracy.

#### 2.3.1. Spatial Attention

In the U-Net decoder, encoder features are passed through skip connections to recover spatial details lost during downsampling. However, not all spatial locations in these features are equally informative for segmentation: background regions and weak textures can propagate noise into the decoding stages.

To mitigate this, we apply a simple, self-gated spatial attention mechanism on the skip features before concatenation. The module learns a per-pixel importance map directly from the skip tensor itself, without conditioning decoder activations or introducing cross-scale gating.

Given a skip feature map $x_{\mathrm{skip}}\in R^{H\times W\times C}$, we compute a single-channel spatial weight map $W\in R^{H\times W\times 1}$ using a 1×1 convolution followed by the sigmoid activation σ:

(1) $$W\;=\;\sigma \left({\mathrm{Conv}}_{1\times 1}\left(x_{\mathrm{skip}}\right)\right)$$

The skip features $x_{\mathrm{skip}}$ are then modulated element-wise by this weight map, resulting in ${x^{\prime }}_{\mathrm{skip}}$:

(2) $${x^{\prime }}_{\mathrm{skip}}\;=\;x_{\mathrm{skip}}\;⊙\;W,$$

where ⊙ denotes element-wise multiplication.

This operation encourages the decoder to focus on spatially important regions—such as vegetation structures—while down-weighting less relevant areas.

#### 2.3.2. Vegetation Global Context Block

To enhance feature representations in regions likely to contain vegetation, we integrate a Vegetation Global Context Block. This module computes a channel-wise attention vector that modulates features based on the estimated vegetation probability, providing a context-aware recalibration akin to Squeeze-and-Excitation networks [46].

Considering H and W, the spatial dimensions of the image, and C the number of channels, given a feature map $F\in R^{H\times W\times C}$ obtained through the convolution of the final encoder features and a vegetation probability map $V\in R^{H\times W}$ with values in the interval [0, 1], calculated using a Sigmoid activated 1×1 convolution of the last encoder stage, the block first computes a soft Masked Global Pooling:

(3) $$w_{c}\;=\;\frac{\sum_{i=1}^{H}\sum_{j=1}^{W}F_{\;i,j,C}V_{\;i,j}}{\sum_{i=1}^{H}\left(\sum_{j=1}^{W}V_{\;i,j}\;+\;\epsilon \right)},\;\forall \;c\;\in \;\left\{1,\;2,\;\ldots ,C\right\},$$

where ϵ is a small constant to avoid division by zero, and $w\in R^{1\times 1\times C}$ is the resulting channel descriptor summarizing feature activations weighted by vegetation likelihood.

The excitation part follows [46]. A channel bottleneck reduces the dimensionality of this descriptor, applies a nonlinearity, and projects it back to the original number of channels to produce a gating vector for each channel $g\in R^{1\times 1\times C}$:

(4) $$g\;=\;\sigma \left(W_{2}\;\mathrm{ReLU}\;\left(W_{1}w\right)\right),$$

where $W_{1}\in R^{\frac{C}{r}\times C}$ and $W_{2}\in R^{C\times \frac{C}{r}}$ are learned 1×1 convolutional weights implementing the dimensionality reduction, and subsequent expansion, by the reduction ratio *r*, chosen so as to divide C, and σ is the sigmoid function. In our case, we have used a reduction ratio of 4.

Finally, the original feature map is weighted channel-wise by the gating vector, obtaining $F^{\prime }$:

(5) $$F^{\prime }\;=\;F\;⊙\;g$$

This operation emphasizes channels that are most relevant to vegetation regions while suppressing less relevant information, effectively conditioning the global feature representation on the predicted vegetation mask.

**Figure 6:**
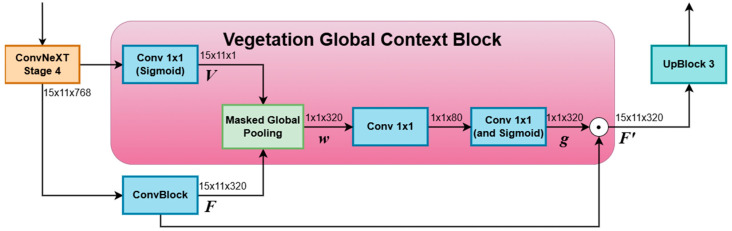
The Vegetation Global Context Block, placed at the bottleneck of the network.

The Vegetation Global Context Block is lightweight, fully differentiable, and is inserted into the bottleneck layer of the encoder–decoder architecture to provide vegetation-aware global context. It is further illustrated in Figure 6.

#### 2.3.3. Deep Supervision

Deep Supervision involves placing auxiliary classification heads at intermediate layers of the decoder. This combats the vanishing gradient problem and forces the intermediate layers to learn semantically meaningful features early in the network. These auxiliary heads are discarded at inference time.

We attach auxiliary heads to the output of each upsampling stage. These output two channels: one for vegetation and the other for crops. This deep supervision serves two purposes. Firstly, it stabilizes optimization by providing direct gradient signals to intermediate decoder layers, mitigating vanishing gradients and accelerating convergence. Secondly, it encourages semantically meaningful representations at multiple spatial scales: coarse decoder stages are guided to capture global vegetation structure, while finer stages progressively refine crop localization. Since vegetation and crop form a hierarchical label structure, supervising both classes at each scale promotes consistent feature disentanglement throughout the decoder rather than deferring all semantic separation to the final layers.

### 2.4. Logical Constraints and Weed Class Inference

Another interesting aspect of our method is the enforcement of logical, hierarchical consistency. Since ideally a pixel cannot represent a crop if it is not part of the vegetation class, we scale the crop predictions by the vegetation probabilities during training. The predicted crop probability $P_{\mathrm{crop}}$ is scaled by the vegetation prediction $P_{veg}$, resulting in ${\hat{P}}_{\mathrm{crop}}$:

(6) $${\hat{P}}_{\mathrm{crop}}={\;P}_{\mathrm{crop}}\;\cdot \;P_{\mathrm{veg}}^{\gamma }$$

where γ = 0.75. This softly suppresses crop predictions in non-vegetation-predicted areas, while leaving room for correction when the vegetation prediction is uncertain.

Subsequently, the Weed probability map $P_{\mathrm{weed}}$ is derived logically rather than predicted independently:

(7) $$P_{\mathrm{weed}}\;=\;\left\{\begin{array}{c} P_{\mathrm{veg}}\;-\;{\hat{P}}_{\mathrm{crop}},\;\;\;\;P_{\mathrm{veg}}\;\geq \;{\hat{P}}_{\mathrm{crop}}\\ \;\;\;\;\;\;\;\;\;\;\;\;\;\;0,\;\;\;\;\;otherwise \end{array}\right.$$

Since the weed probability map is obtained through subtraction, its values tend to be lower in magnitude compared to directly predicted logits. For this reason, we apply a slightly lower decision threshold during binarization to recover the final weed mask. Operating in probability space allows uncertainty from both vegetation and crop predictions to be preserved, particularly around boundaries and ambiguous regions. In preliminary experiments, this soft formulation consistently outperformed a hard logical XOR between vegetation and crop masks, which enforces strict binary separation and proved more sensitive to misclassifications and boundary noise.

### 2.5. Loss Function

The total objective function, $L_{\mathrm{total}}$, is a weighted sum of the primary head losses and the auxiliary deep supervision losses. In the following equations, we will consider *P* and *G* flattened maps of dimension N = HW (where H is the initial height, and W the width), with i indexing pixels.

We utilize the Dice Loss ($L_{\mathrm{Dice}}$) to handle class imbalance by maximizing the overlap between the predicted probability map P and the ground truth G:

(8) $$L_{\mathrm{Dice}}(P,\;G)\;=\;1\;-\;\frac{2\cdot \sum_{i=1}^{N}P_{i}G_{i}}{\sum_{i=1}^{N}P_{i}+\sum_{i=1}^{HW}G_{i}}$$

By weighting the contribution of each pixel relative to the sum of predicted and ground truth pixels, Dice Loss emphasizes overlap rather than absolute pixel counts, which makes it particularly effective when foreground regions are small compared to the background. To penalize boundary inaccuracies, we incorporate a Boundary Dice Loss, $L_{\mathrm{bound}}$. We compute edge maps for both the prediction and ground truth using convolution with a discrete Laplacian kernel K (approximate second-order derivative). The loss is computed as the Dice loss between these edge maps:

(9) $$\begin{array}{c} L_{\mathrm{bound}}(P,\;G)\;=\;1-\mathrm{Dice}\left(\mathrm{ReLU}\left(P*K\right),\;\mathrm{ReLU}\left(G*K\right)\right),\\ K\;=\left[\begin{array}{ccc} 1 & 1 & 1\\ 1 & -8 & 1\\ 1 & 1 & 1 \end{array}\right] \end{array}$$

The application of the Rectified Linear Unit (ReLU) function is essential because the Laplacian kernel naturally produces a zero-crossing at edges, resulting in both positive and negative values. Since the Dice coefficient is a set-similarity metric undefined for negative inputs, the ReLU operation eliminates the negative component—typically corresponding to the internal side of the boundary—and isolates the positive external contour. This ensures numerical stability and converts the raw derivative into a clean, non-negative edge mask, allowing the network to strictly enforce geometric alignment between the predicted and ground truth boundaries.

For the Crop head, we also utilize Binary Cross-Entropy (BCE) to strictly penalize pixel-level misclassifications, ensuring that all pixels within the object region, not just its boundaries, are predicted confidently. Unlike Dice, which considers the image as a set, BCE evaluates the confidence of each pixel independently and acts as a stabilizing term, ensuring that the model learns the general distribution of pixels early in training before fine-tuning the boundaries.

(10) $$L_{\mathrm{BCE}}(P,\;G)=-\frac{1}{N}\sum_{i=1}^{N}[G_{i}\;\cdot \;\log(P_{i})+(1-G_{i})\;\cdot \;\log(1-P_{i})],$$

To further enforce the logical consistency of the crop class and improve the recall of its prediction within vegetated areas, we have found it beneficial to also include an $L_{\mathrm{CropRecall}}$ term, which represents the Dice loss computed exclusively over pixels labeled as vegetation, and encourages the network to recover all crop pixels within the broader vegetation mask:

(11) $$L_{\mathrm{CropRecall}}({\hat{P}}_{\mathrm{crop}},\;G_{\mathrm{crop}},{\;G}_{\mathrm{veg}})\;=\;L_{\mathrm{Dice}}\left({\hat{P}}_{\mathrm{crop}}\;\cdot \;G_{\mathrm{veg}},\;G_{\mathrm{crop}}\;\cdot \;G_{\mathrm{veg}}\right)$$

The final loss functions for the Vegetation and Crop heads combine Dice, Binary Cross Entropy (BCE), and Boundary Dice terms. The total loss is calculated as:

(12) $$L_{\mathrm{veg}}\;=\;L_{\mathrm{Dice}}\left(P_{\mathrm{veg}},\;G_{\mathrm{veg}}\right)\;+\;\lambda_{\mathrm{bV}}L_{\mathrm{Bound}}\left(P_{\mathrm{veg}},{\;G}_{\mathrm{veg}}\right)$$

(13) $$\begin{array}{c} L_{\mathrm{crop}}=\mathrm{BCE}\left({\hat{P}}_{\mathrm{crop}},\;G_{\mathrm{crop}}\right)+L_{\mathrm{Dice}}\left({\hat{P}}_{\mathrm{crop}},\;G_{\mathrm{crop}}\right)+\lambda_{\mathrm{bC}}L_{\mathrm{Bound}}\left({\hat{P}}_{\mathrm{crop}},{\;G}_{\mathrm{crop}}\right)+\\ \;0.5\;\cdot \;L_{\mathrm{CropRecall}}({\hat{P}}_{\mathrm{crop}},{\;G}_{\mathrm{crop}},{\;G}_{\mathrm{veg}}) \end{array}$$

(14) $$L_{\mathrm{weed}}={0.7\;\cdot \;L}_{\mathrm{Dice}}\left(P_{\mathrm{weed}},{\;G}_{\mathrm{weed}}\right)+\lambda_{\mathrm{bW}}L_{\mathrm{Bound}}\left({\hat{P}}_{\mathrm{weed}},{\;G}_{\mathrm{weed}}\right)$$

(15) $$L_{\mathrm{total}}=\lambda_{V}L_{\mathrm{veg}}+\lambda_{C}L_{\mathrm{crop}}+\lambda_{W}L_{\mathrm{weed}}+\sum_{k=1}^{3}\lambda_{\mathrm{aux}}L_{\mathrm{aux}}^{\left(k\right)}$$

where λ terms are hyperparameters weighting the contribution of each component, and $L_{\mathrm{aux}}^{\left(k\right)}$ represents the Dice loss from the k-th deep supervision head.

## 3. Results

We have built our U-Net architecture with different ImageNet-pretrained encoders using the Pytorch and timm libraries. As the patches were non-overlapping and the campaigns included different weather, soil conditions, and stages of growth, we chose to split the whole dataset for training, validation, and testing. After excluding the second campaign, the images were randomly divided into 80% training data (1267 patches), 10% validation data (158 patches), and 10% testing data (159 patches). Each image was standardized by the ImageNet dataset’s mean and standard deviation.

For every experiment, we used the AdamW optimizer, and a Cosine Annealing learning rate scheduler was also employed. Each model was trained for a total of 50 epochs, and only the checkpoint where the model obtained the best validation score was saved. Most hyperparameters were chosen after a grid search. We have grouped them in Table 1.

The experiments have been performed on a personal computer with an i5-9300H CPU, a GTX 1660TI GPU, and 8 GB of RAM.

During model validation, we evaluated multiple binarization thresholds applied to the final probability maps produced by the model. The results have shown that better scores were consistently obtained with lower steps, something to be expected as the weeds class probabilities are obtained as a subtraction. This behavior can also be attributed to the characteristics of the segmentation task: crop and weed boundaries are often ambiguous, partially occluded, or affected by illumination variability, leading to moderately confident predictions in boundary and fine-structure regions. A higher binarization threshold tends to suppress these low- to mid-confidence activations, fragmenting predicted regions and increasing false negatives, particularly for thin or early-stage weeds. In contrast, a lower threshold preserves weaker but spatially coherent activations, resulting in more complete object representations and improved recall.

We chose to apply a 0.3 threshold for our final models, and we report the scores with this parameter in Table 2. The scores include the IoU, mIoU over the three classes, Dice, Overall Pixel Accuracy, and Overall Cohen’s Kappa coefficient. The inference speed was determined by measuring the total elapsed time of the batch-wise iteration loop over the test set, beginning immediately prior to the first data fetch and concluding after the final GPU synchronization, without any warm-up. By timing the complete execution cycle, the reported throughput accounts for the entire processing pipeline, including the initial reading of the image file, resizing and normalization, and the post-processing step of thresholding. For [38] we note the average scores reported in the paper across all the campaigns except for the second one, which we did not use. The inference time is also the one reported in the respective paper, so it has been measured on a different system than the other cases. For [27] we report their stated results on a split of 40 test images. The paper does not explicitly mention whether the metrics are measured on all three classes or just on crops and weeds. The inference speed is calculated based on their reported time of 35 ms for an image, and it has not been measured on the same system as the others.

To test our architecture against a heavyweight state-of-the-art segmentation model, we have also fine-tuned a pre-trained Mask2Former [47] model with a SwinTransformer-Tiny backbone in the same conditions, on the 480 × 352 patches, for 50 epochs. Mask2former is an architecture designed as a universal segmentation model, capable of addressing all segmentation tasks, and is based around a Transformer decoder with masked attention. It has a much higher parameter count and computational cost than our models. The results are presented in Table 3. The metrics show that our architecture achieves higher performance, with a much faster inference speed.

## 4. Discussion

The quantitative results in Table 2 reveal consistent trends across backbone families and input resolutions. As expected, higher input resolutions generally lead to improved weed segmentation performance, reflected by both IoU and Dice scores, confirming that weeds, even though they are not directly predicted, due to their thin, fragmented, and irregular morphology, benefit more strongly from increased spatial detail than crops. This effect is visible for ConvNeXt V2 Tiny and FastViT variants, where downsampling to 256 × 256 results in a noticeable degradation of weed IoU and Dice. Interestingly, crop segmentation performance is greater for the lower resolution inputs. We believe this occurs because downsampling smooths the image, which aids the segmentation of the relatively uniform and regular shapes of the tobacco plants. Across all evaluated backbones, crop segmentation achieves higher IoU and Dice scores than weed segmentation. This gap is expected, given the greater visual variability and boundary ambiguity of weed regions. Importantly, the relatively strong weed performance observed even with lightweight backbones suggests that deriving weeds implicitly as vegetation excluding crops helps stabilize predictions, reducing spurious weed activations in non-vegetated areas and improving boundary coherence.

From an efficiency standpoint, FastViT and RepViT architectures achieve substantially higher throughput, exceeding 40 images per second at higher resolutions and over 60 images per second at lower resolutions, making them well-suited for real-time deployment. ConvNeXt V2 Tiny, while slower, consistently delivers the strongest weed segmentation accuracy, indicating a trade-off between representational capacity and computational cost. MambaVision-T occupies an intermediate position, offering balanced performance across both accuracy and speed.

Overall, the results demonstrate that the proposed single-stage architecture, jointly predicting crop and vegetation masks and deriving weed regions as residual vegetation, generalizes well across diverse encoder designs. This flexibility allows the architecture to be paired with either accuracy-oriented or speed-oriented backbones, without sacrificing logical consistency. Compared with the other two approaches on the same dataset, this method achieves at least more than double the inference speed while maintaining comparable—or in some cases superior—Dice and IoU scores, as demonstrated by the ConvNeXt V2 Tiny backbone at full patch resolution (see Table 2).

**Figure 7:**
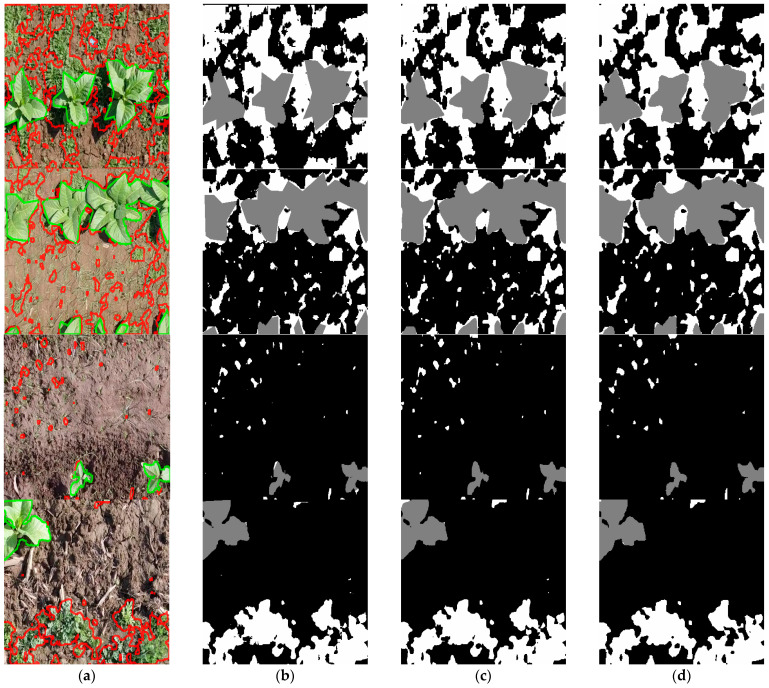
Examples of segmentation masks generated by the proposed architecture with two different backbones at different input resolutions: (**a**) Original image with the ground-truth mask’s edges overlaid (green for tobacco, red for weeds); (**b**) Ground-truth mask; (**c**) Predicted mask with the Conv-NeXT V2 encoder (480 × 352 input resolution); (**d**) Predicted mask with the FastViT S12 encoder (256 × 256 input resolution).

Figure 7 presents a few examples of predicted segmentation masks, along with the original images overlaid with the ground-truth segmentation masks’ edges (Figure 7a) and the ground-truth masks (Figure 7b), using both the most accurate backbone (ConvNeXt V2 Tiny at full patch resolution) (Figure 7c) and the fastest (FastViT S12 at 256 × 256 resolution) (Figure 7d). Both models show great promise in their ability to distinguish between the two classes of interest and to delineate their shapes.

Looking at the ground-truth masks, we may notice that some of them have shapes with rough, sharp angles that are not entirely consistent with the actual plants in the image. This inconsistency may affect the learning and evaluation of our models: while it is true and visibly clear that the ConvNeXt V2 model has the higher scores, since it resembles the ground-truth better, there are instances where the theoretically less precise FastViT model may actually be closer to reality, as is the case in the first sample (row 1 in Figure 7).

The last two examples (rows 3 and 4 in Figure 7), in contrast, highlight the limitations of the lower-resolution FastViT model in capturing very small weed structures. These observations underline that the reported quantitative performance should be interpreted in the context of the possible limitations of the ground-truth annotations.

While the proposed single-stage architecture prioritizes real-time execution and logical consistency for edge deployment, it is important to contextualize this approach alongside recent advancements in foundation models, such as the Segment Anything Model 3 (SAM3) [48]. As demonstrated by our benchmarking against Mask2Former (Table 3), heavy transformer-based segmentation models incur significant computational costs (46.05 GFLOPs) and reduced inference speed (4.47 images/second). SAM3, representing a state-of-the-art paradigm in zero-shot, generalized segmentation, features a much larger parameter count and relies on iterative, prompt-driven interfaces. This results in even higher computational demands, which may limit its suitability for integration in resource-constrained agricultural platforms. Furthermore, our proposed hierarchical formulation produces stable predictions without the need for the manual visual or text prompts required by such foundational approaches. Therefore, while models such as SAM3 offer strong potential for large-scale offline agronomic analysis, particularly in scenarios with limited or no annotations, lightweight, task-specific and fully supervised architectures, such as the one proposed in this study, remain well-suited for practical agricultural applications like automated pesticide dispersion.

## 5. Conclusions

This paper has presented a U-Net architecture for the segmentation of tobacco crops and weeds. The architecture incorporated custom modules, such as the Vegetation Global Context Block, and a dual-headed output that separately predicts vegetation and crop masks. Logical constraints are enforced both during training and inference, with weed regions derived hierarchically as the difference between vegetation and crop predictions. Several modern backbones have been tested, and experimental results demonstrate that the proposed approach achieves improved segmentation accuracy while also reducing inference time compared to previous methods evaluated on the same dataset. Although prior work has explored similar differential formulations, these are typically implemented using multi-stage pipelines, which introduce additional complexity and latency. In contrast, our approach integrates the full hierarchical reasoning within a single end-to-end model, enabling efficient inference without sacrificing performance.

Future work will focus on improving spatial feature selection through more expressive attention mechanisms, as well as extending the hierarchical segmentation framework to multi-crop or multi-weed species scenarios. Robustness across domains and acquisition conditions represents another important direction for further experimentation. The proposed methods should be systematically evaluated across diverse environments, including different crop types, growth stages, weed density, soil compositions, illumination conditions, and weather scenarios, to assess their generalization capabilities. Variations in sensor characteristics, image resolution, and acquisition platforms should also be considered, as they may significantly impact model performance. Techniques such as domain adaptation, data augmentation, and multi-domain training could be explored to mitigate these effects.

The ultimate objective is the integration of the proposed model into a real-time, automated pesticide dispersion system, which may operate as part of a larger agricultural monitoring platform. In such a setting, the model must meet strict constraints in terms of inference speed, computational efficiency, and reliability, enabling precise, site-specific weed treatment. This would allow for targeted pesticide application, reducing chemical usage, minimizing environmental impact, and improving overall agricultural sustainability.

## Figures and Tables

**Table 1:** Hyperparameters used for the training of the models, selected through a grid search.

Hyperparameter	Value
$\lambda_{\mathrm{bV}}$	0.3
$\lambda_{\mathrm{bC}}$	0.05
$\lambda_{\mathrm{bW}}$	0.3
$\lambda_{\mathrm{aux}}$	0.05
$\lambda_{V}$	1.3
$\lambda_{C}$	1.6
$\lambda_{W}$	1
Learning Rate	1 × 10^−4^
Weight Decay	1 × 10^−4^

**Table 2:** Results obtained with the different backbones we experimented with in our architecture, along with 2 other works on the same dataset. Bold was used for the best parameter values.

Backbone	Resolution	IoU Crops	IoU Weeds	mIoU	Dice Crops	Dice Weeds	Overall Accuracy	Overall Kappa	Inference Speed (Im/s)
[38]	480 × 352	0.7460	0.7800	-	-	-	-	-	1.428
[39]	320 × 320	0.8894	0.8582	-	0.9374	0.9215	-	-	10.79
[27]	480 × 352	-	-	0.8648	-	-	0.9208	-	28.57
ConvNeXt V2 Tiny	480 × 352	**0.8990**	**0.8843**	**0.9225**	0.9424	**0.9372**	**0.9790**	**0.9586**	22.90
	256 × 256	0.8971	0.8366	0.9002	**0.9446**	0.9086	0.9695	0.9400	35.46
Mamba Vision-T	480 × 352	0.8930	0.8498	0.9046	0.9392	0.9165	0.9718	0.9444	24.06
FastViT_S12	480 × 352	0.8919	0.8459	0.9031	0.9389	0.9141	0.9713	0.9434	44.52
	256 × 256	0.8914	0.8197	0.8916	0.9412	0.8982	0.9665	0.9339	**64.81**
FastViT_SA24	480 × 352	0.8932	0.8536	0.9067	0.9392	0.9190	0.9728	0.9463	25.72
RepViT-M2	480 × 352	0.8942	0.8507	0.9058	0.9401	0.9171	0.9724	0.9454	47.77

**Table 3:** Comparison of our best-performing model with a fine-tuned Mask2Former architecture. Bold was used for the best parameter values.

Architecture	No. Params	FLOPs	IoU Crops	IoU Weeds	Dice Crops	Dice Weeds	Inference Speed (Images/s)
Mask2former	47.40 M	46.05 G	0.8918	0.8686	0.9373	0.9281	4.47
Ours, with ConvNeXt V2 Tiny	32.52 M	17.93 G	**0.8990**	**0.8851**	**0.9424**	**0.9376**	**22.90**

## Data Availability

The original contributions presented in this study are included in the article. The corrected dataset variant and the test split we used are available in Zenodo at 10.5281/zenodo.19422418. Further inquiries can be directed to the corresponding author.
